# MicroRNA-218 Regulates Signaling Lymphocyte Activation Molecular (SLAM) Mediated Peste des Petits Ruminants Virus Infectivity in Goat Peripheral Blood Mononuclear Cells

**DOI:** 10.3389/fimmu.2019.02201

**Published:** 2019-09-20

**Authors:** Xuefeng Qi, Ting Wang, Zhen Li, Yangli Wan, Bo Yang, Wei Zeng, Yanming Zhang, Jingyu Wang

**Affiliations:** College of Veterinary Medicine, Northwest A&F University, Yangling, China

**Keywords:** MicroRNA-218, SLAM, PPRV, goat PBMCs, innate immune response, hemagglutinin protein

## Abstract

Peste des petits ruminants virus (PPRV) has emerged as a significant threat to the productivity of small ruminants worldwide. SLAM was identified as the primary receptor for PPRV and other *Morbilliviruses*, although the regulation of SLAM expression is not yet fully understood. In this study, we revealed a novel mechanism by which PPRV upregulates its receptor SLAM expression and thereby benefits its replication via suppressing miR-218, a novel negative miRNA directly targeting *SLAM* gene. We demonstrated that PPRV infection downregulates miR-218, which in turn enhances SLAM expression on the surface of goat peripheral blood mononuclear cells (PBMCs), thus promoting PPRV replication. Since SLAM signaling may modulate the immune responses induced by PPRV infection, we further examined the effect of SLAM expression on the production of various cytokines by PBMCs in the absence or presence of PPRV. We demonstrated that miR-218-mediated SLAM expression modulates the expression of IFN-γ, TNF-α, and IL-10, importantly, these modulatory effects were enhanced in the presence of PPRV infection. Furthermore, our data clearly showed that PPRV H protein is sufficient to regulate miR-218-mediated SLAM expression. Taken together, our results suggest a novel mechanism involving post-transcriptional regulation of SLAM receptor expression on goat PBMCs during PPRV infection.

## Introduction

Peste des petits ruminants virus (PPRV) is a member of the Morbillivirus family and is one of highly contagious fatal diseases known to domestic and wild small ruminants (1–3). Goat is naturally more susceptible to PPRV than sheep due to the host- or virus-derived factors (1, 4). PPRV infection usually caused severe suppression of immune responses in goat, which favors secondary infections (2, 3, 5). A predominant Th2 response induced by PPRV infection account for the suppression of cellular immunity (6, 7), although virus-specific immunity is efficiently stimulated (8, 9). The PPRV vaccine strain, Nigeria 75/1, has been shown to protect against virus isolates of all four lineages in most countries (10, 11). Although the vaccine with attenuated PPRV has been found to be highly efficacious, safe, and potent in small ruminants, it has been showed that the vaccine virus induced an early and transient lymphopenia in goats (6). Importantly, transient immune suppression to other antigens in goats immunized with PPRV vaccine was found (6).

Signaling lymphocyte activation molecules (known as SLAM or CD150) expressed on the surface of lymphocytes act as receptor for PPRV entry (12). SLAM signaling has been reported to function as a modifier in immunodeficiency disease (13–15). Previous studies have indicated that SLAM signaling may play a key role in mediating the strong immunosuppression induced by the measles virus (7, 16). In addition, the interaction between SLAM and PPRV has been implicated (12, 17, 18), and recent studies have demonstrated that H protein of PPRV can bind SLAM receptor (19). Thus, regulation of SLAM receptor might mediate PPRV-induced immunosuppression.

MicroRNAs (miRNAs) play a key role in regulation of gene expression post-transcriptionally (20–22). A growing number of reports suggest that virus infection caused significant change of cellular gene expression profile by altering cellular miRNAs expression (23–25). To date, the number of identified miRNAs in goat is only 420 exist in the Sanger miRBase v22.0 (March 2018). Our recent study has identified 103 known and 213 novel miRNAs from PPRV-infected goat PBMCs by using small RNA deep sequencing (26). We focused on novel miR-218 by TargetScan to target *SLAM* gene and its fold change. We found that PPRV infection stimulates a rapid increased SLAM expression at early infection followed by a gradual decreased expression. Importantly, an inverse correlation between the expression of miR-218 and SLAM during PPRV infection was observed. Increased SLAM expression mediated by miR-218 at early PPRV infection facilitates the virus entry into host cells and subsequently replication. To the best of our knowledge, this study is the first to show that SLAM expression is involved in post-transcriptional regulation during PPRV infection.

## Materials and Methods

### Animals

Healthy 6 months old goats were obtained from the Experimental Animal Center of the Northwest A&F University (Yangling, China) and were housed in appropriate containment facilities and had *ad libitum* access to feed and water. Goats were screened for PPRV antibodies using competitive ELISA and serum neutralization test and both showed negative. All experiments were performed in accordance with local animal welfare regulations.

### PBMCs Isolation and Virus Infection

Goat PBMCs were isolated using Histopaque-1077 (Sigma, USA) by density gradient centrifugation following the manufacturer's instructions. Then, isolated cells from each goat were suspended into 70 ml RPMI-1640 medium (Hyclone, Logan, UT, USA) supplemented with 10% fetal calf serum (FCS), 100 mg/ml penicillin, and 100 IU/ml streptomycin. The PPRV vaccine strain, Nigeria 75/1, was obtained from the Lanzhou Veterinary Research Institute, Chinese Academy of Agricultural Sciences (Lanzhou, China). Virus stock was prepared by collecting the infected Vero cell supernatant when cytopathic effect (CPE) affected about 80% of the cells. The virus was harvested by three cycles of freezing and thawing and stored at −80°C and purified by banding on sucrose gradient. The purified virus titers were estimated by estimating 50% tissue culture infective doses (TCID_50_) using Vero cells in 96 well micro titer plate. The purified virus was tested for its infectivity in the green monkey kidney cell line (Vero) obtained from American Type Culture Collection (ATCC) and was used further for infection in goat PBMCs.

For virus infection, goat PBMCs were seeded into six well plates at a density of 1 × 10^5^ cells/ml and were inoculated with Nigeria 75/1 at a multiplicity of infection (MOI) of 1.0. After 1 h of adsorption, infected cells were maintained in RPMI-1640 medium (Hyclone, Logan, UT, USA) supplemented with 2% FCS. PBMCs inoculated with similarly purified preparation from triple freeze-thawed Vero cells were used as the mock-infected group. Viral infection in PBMCs was confirmed with CPE, one-step growth curve and Western blot. The CPE were observed under a light microscope at 0, 24, 48, and 72 hpi. Western blot was performed using a polyclonal antibody against PPRV N protein to determine virus replication at the different time points after infection. Three replicates of PPRV- and mock-inoculated cultures were prepared at indicated time point.

### Flow Cytometric Assay

The changes of SLAM expression cells among goat PBMCs over 48 h transfected with different concentrations of miR-218 mimic or inhibitor in comparison with the negative controlled cells were analyzed by flow cytometry. Briefly, cells (1 × 10^6^) were incubated with FITC-conjugated anti-SLAM antibody (Invitrogen) as described by the manufacturer's instruction. For the detection of intracellular PPRV protein, cells were incubated with a mouse anti-PPRV-N monoclonal antibody provided by China Animal Health and Epidemiology Center (Qingdao, China), washed three times, and then stained with PE-conjugated anti-mouse IgG antibody. Then, the cells were washed twice in cold phosphate buffered saline (PBS) and analyzed on a FACSCalibur (BD Biosciences, San Jose, CA). For evaluation of apoptosis, the cells were harvested, wash thrice with PBS, centrifuged, and suspended in 500 μl of 10 × binding buffer, followed by treatment with 10 μl of FITC-labeled annexin V per sample for 10 min at room temperature. Then, the infected cells were stained with 5 μl of propidium iodide (PI) per sample for 5 min, followed by analysis on a FACSCalibur (BD Biosciences, San Jose, CA). Annexin V-positive and PI-negative cell populations in the lower right quadrant of the Annexin V vs. PI FACS plots were considered apoptotic cells.

### Western Blot Analysis

Protein homogenates from goat PBMCs were extracted as previously described (27). Briefly, the cells were lysed for 20 min on ice in ice-cold lysis buffer (Roche). The lysates were centrifuged at 12,000 × g for 20 min at 4°C to obtain a clear lysate. The protein content of each sample was determined using the BCA Protein Assay Kit (Thermo Scientific). Then, equal amounts of protein were separated on a 12% SDS-polyacrylamide gel and transferred to polyvinylidene difluoride membranes. Membranes were probed overnight at 4°C with an anti-PPRV-N monoclonal antibody provided by China Animal Health and Epidemiology Center (Qingdao, China), or a rabbit polyclonal antibody against sheep SLAM (1:2000; Santa Cruz). The bands were visualized using horseradish peroxidase (HRP)-conjugated goat anti-mouse IgG (1:15000, Boster) or goat anti-rabbit IgG (1:20000, Boster) prior to the ECL protocol (Amersham Biosciences, Piscataway, NJ, USA). As an internal standard, all membranes stripped with primary antibodies were reprobed with anti-β-actin antibody (Invitrogen). Changes in protein expression were determined after normalizing the band intensity of each lane to that of β-actin. Signal was visualized using the Konica SRX 101A developer (Konica Minolta Medical Imaging, Wayne, NJ, USA) and the Quantity One software (Bio-Rad, Mississauga, ON, Canada) was used to densitometrical analysis.

### Confocal Immunofluorescence Microscopy

Goat PBMCs grown on coverslips were infected with PPRV at an MOI of 1. At the indicated times post-infection, the cells were washed four times with PBS and fixed in 4% paraformaldehyde. The cells were washed again four times with PBS and treated with 0.1% Triton X-100 for 15 min. The cells were then incubated with 1% bovine serum albumin (BSA) and the appropriate primary antibodies for 1 h at 37°C. Then, the cells were washed and incubated simultaneously with FITC- or Cy3- conjugated secondary antibodies. Finally, the cells were treated with a Hoechst 33342 solution for 5 min and analyzed under a confocal microscope (CLSM Leica SP8, Germany).

### Illumina Small RNA Deep Sequencing

Goat PBMCs were infected with PPRV at an MOI of 1 or mock infected for 24 h, and then the cells were harvested. Total cellular RNA was extracted using TRIzol reagent (Invitrogen, Waltham, MA, USA) according to the manufacturer's protocol. The quality and quantity of RNA were evaluated by using an Agilent 2100 Bioanalyzer and Agilent RNA 6000 Nano kit, and then the RNAs were given to Annoroad Gene Technology (Beijing, China) for small RNA deep sequencing using an Illumina HiSeq 2000 instrument. The data then were analyzed by Annoroad Gene Technology. The bioinformatics tools RNAhybrid, TargetScan, miRBase, and MiRanda were utilized to determine potential target genes of differential expressed miRNA.

### RNA Isolation and Real-Time PCR Analysis

Total RNA was extracted from goat PBMCs using TRIzol reagent (Invitrogen) according to the manufacturer's instructions. RNA was then reversed using Superscript III (Invitrogen) and random primers (Invitrogen). Real-time quantitative PCR was carried out using an ABI 7500 System (Applied Biosystems, Warrington, UK) and Power SYBR Green PCR Master Mix (Applied Biosystems). The sequences of the primers used were as follows: *SLAM*, 5′-AACTGGAGTGAGGAAGCAGGT-3′ (forward), 5′-CGCAATGCAGATGTAGACGTT-3′ (reverse); β*-actin*, 5′-AGACATCAGGGTGTGATGGTTGGT-3′ (forward), 5′-TGGTGACAATACCGTGTTCAATGG-3′ (reverse). The PCR cycling conditions were 20 s at 95°C; followed by 40 cycles of 3 s at 95°C and 30 s at 60°C. Expression of β-actin was used to normalize cDNA levels for differences in total cDNA levels in the samples. Then, the Ct (*d*) was used to calculate the fold difference in copy number using the formula f = 2^(−d)^, where f = the fold difference in the expression of a specific gene and d = the difference in the Ct values between the compared sources of mRNA (corrected for differences in the β-actin levels). We normalized each sample to control cell sample #1. Melt curves were performed to confirm the purity of the amplified products.

Goat cytokines gene mRNA expression levels were also detected and the sequences of the primers and reaction conditions have been described previously (28).

For detection of miR-218, total RNA was reverse transcribed and quantitative real-time RT-PCR analysis was performed using Bulge-loop^TM^ miRNA qRT-PCR Primer Sets (one RT primer and a pair of qPCR primers for each set). The primers for miR-218 and internal standard 5S snRNA are designed by RiboBio Inc. (GuangZhou. China) and the sequences are covered by a patent. Briefly, 2 μl of cDNA was added to 10 μl of the 2 × SYBR green PCR master mix with 0.2 μl of Taq polymerase enzyme (RiboBio, China), 200 nM of each primer and ddH_2_O to a final volume of 20 μl. The reactions were amplified for 2 s at 95°C and 20 s at 60°C for 40 cycles. The thermal denaturation protocol was run at the end of the PCR to determine the number of products that were presented in the reaction mix. Reactions were typically run in duplicate. Micro RNA relative expression quantity was detected and calculated using relative quantitative standard curve method.

### Transient Transfection of miRNA

Goat PBMCs were grown to logarithmic phase in six well plates with antibiotic-free medium the day before transfection. The miRNA transfection, including miR-218 mimic, mimic control (MC), miR-218 inhibitor, and inhibitor control (NC) was performed with Lipofectamine RNAiMAX (Life Technologies, USA) on cells of 50% confluence according to the manufacturer's protocol. The final concentrations of miR-218 mimic, miR-218 inhibitor or their negative controls (RiboBio, GuangZhou. China) was 100 nM. The effect of transfection was examined by quantitative RT-PCR and Western blot.

### Dual-Luciferase Reporter Assay

HEK293 cells were transfected with 10 ng each of psiCheck2 reporter plasmids along with 15 pmol of the miR-218 mimic or an identical amount of the negative control with Lipofectamine 2000 (Invitrogen). After 48 h, the cells were lysed, and the firefly and Renilla luciferase activities were measured with the Dual-Luciferase Reporter Assay System Kit (Promega, Madison, USA) according to the manufacturer's protocol. Each fragment containing the putative miRNA-binding sites was cloned in psiCheck2 immediately downstream of the gene encoding renilla luciferase by the Protein Expression Laboratory (SAIC, Frederick, MD). The results are presented as the ratio of Renilla luciferase activity to firefly luciferase activity. Each transfection was performed at least three times and was assayed in triplicate.

### Virus Titration

Virus progeny production was determined by titration as described previously (29). The viral supernatants from goat PBMCs were collected at the indicated time points after virus inoculation, and the TCID_50_ was calculated by the Reed-Muench method.

### Plasmids Construct, Virus Protein Expression, and Purification

PPRV genes V, H, and N were amplified from PPRV genomic cDNA and cloned into pCDNA3.1(+) (Invitrogen, V790-20) or pET30a vectors (Novagen,69909-3). His-V, His-H, His-N were expressed in *E.coli* BL-21 and purified using Ni-NTA resin. The purified proteins refolding was achieved by gradient dilution from guanidine hydrochloride solution, and desalinated by ultrafiltration. After purification and renaturation, three proteins exist in soluble form with the concentration 3.256–4.593 mg/mL and the homogeneity 96.71%. The three purified proteins were then detected using western blotting with the corresponding antibody.

### Statistical Analysis

All values are expressed as the arithmetic means of triplicates ± standard deviation (SD). Significance was determined by a one-way ANOVA with a Dunnett-post test, or by the Student paired *t*-test. Values of *P* < 0.05 were considered statistically significant.

## Results

### PPRV Infection Alters the Expression Levels of SLAM in Goat PBMCs

**Figure 1:**
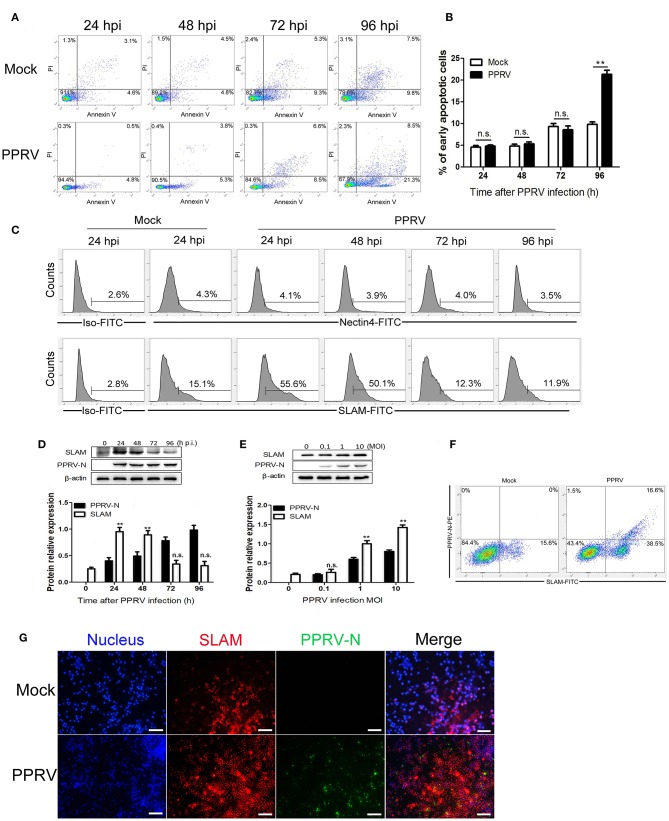
Determination of the expression of SLAM during PPRV infection in goat PBMCs. **(A)** Goat PBMCs were infected with PPRV at a multiplicity of infection (MOI) of one for the indicated times, the cell samples were dual-labeled with Annexin V and PI and analyzed by flow cytometry. **(B)** Proportions of apoptotic cells in **(A)**. **(C)** Goat PBMCs were infected with PPRV at a MOI of 1 for the indicated times, the cell-surface expression of SLAM on goat PBMCs was measured by flow cytometry. **(D,E)** Goat PBMCs were infected with PPRV at a MOI of 1 for the indicated times **(D)** or at different MOIs for 24 h **(E)**, and the protein levels of SLAM and PPRV-N expression were measured by Western blot. **(F,G)** Goat PBMCs were infected with PPRV at a MOI of 1 for 24 h, the expression of SLAM on goat PBMCs was measured by flow cytometry **(F)** and immunofluorescent staining **(G)**. **(G)** Representative fluorescence photomicrographs shows the co-localization of SLAM (red) and PPRV-N (green) in goat PBMCs. Cell nuclei were labeled with Hoechst 33342. Scale bars = 50 μm. Results are expressed as means ± standard deviation (SD) from three independent experiments. *P*-values were calculated using Student's t test. An asterisk indicates a comparison with the indicated control. *P < 0.05, **P < 0.01.

We first sought to determine the effect of PPRV infection on apoptosis induction in goat PBMCs, and the infected cells were collected and subjected to flow cytometry analysis with fluorescein isothiocyanate (FITC)-Annexin V and propidium iodide (PI). The time course of apoptotic cells analysis in mock- and PPRV-infected PBMCs was showed in Figures 1A,B. Collectively, there was not different for the percentage of apoptotic cells between virus-infected and mock-infected cells within 72 h post infection (hpi). However, more apoptotic cells were detected in PPRV-infected groups at 96 hpi than in mock-infected groups (*P* < 0.05).

PPRV is characteristiced by receptor-dependent lymphotropism and epitheliotropism. PPRV receptor tropism to CD46, SLAM/CD150, and PVRL4/nectin4, has been well-documented (12, 17, 19, 30). To determine whether Nectin4 and SLAM expression in goat PBMCs is modulated after PPRV infection, FACS analysis of Nectin4 and SLAM surface expression was performed. Nectin4 expression was not altered during PPRV infection as compared to mock infected cells within 96 hpi (Figure 1C). However, infection of PPRV caused a rapid increase in SLAM cell surface expression at 24 hpi followed by a decreased expression (Figure 1C).

PPRV enters lymphoid cells through cell surface expressed SLAM receptor (17, 19, 26). To further determine SLAM expression levels and PPRV replication in goat PBMCs, SLAM protein and PPRV N protein expression were analyzed by Western blot. Analogous to our results obtained from FACS analysis, an increased SLAM expression levels was observed at 24 hpi followed by a progressively decreased expression (Figure 1D). Additionally, higher levels of SLAM were detected in the cells infected with PPRV at a higher MOI (Figure 1E). Furthermore, a persistently increased PPRV replication levels was detected in a post infection time- and virus dose-dependent manner (Figures 1D,E). Since PPRV infection caused a rapid increase in SLAM expression at 24 hpi, we also detected SLAM expression in individual infected and non-infected cells by FACS at 24 hpi, in combined with intracellular staining for PPRV N protein. As show in Figure 1F, a significant increased SLAM expression was detected on the surface of both PPRV positive and PPRV negative staining cells in PPRV infected group as compared to mock group. Importantly, PPRV infection caused a significant increased percentage of SLAM+PPRV- cells as compared with mock infected cells. Similar to the results from FACS analysis, immunofluorescence detection showed that weak staining of SLAM was detected in mock infected cells, conversely, strong SLAM immunostaining was observed in PPRV infection group at 24 hpi, as compared with mock infection group (Figure 1G). Interestingly, very strong SLAM staining was observed in PPRV negative staining cells in PPRV infection group as compared with mock infection group (Figure 1G).

### miR-218 Targets the 3′UTRs of SLAM

**Figure 2:**
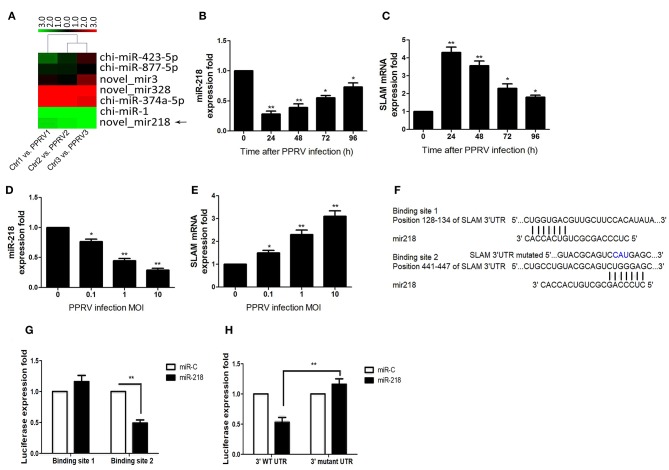
Direct targeting of the 3′UTRs of SLAM mRNA by miR-218. **(A)** Heatmap of miRNAs predicted targets SLAM family members in PPRV-infected goat PBMCs versus mock-infected cells. **(B–E)** Goat PBMCs were infected with PPRV at an MOI of 1 for the indicated time points **(B,C)**, or at different MOIs for 24 h **(D,E)**, and the expression of miR-218 **(B,D)**, and SLAM mRNA **(C,E)** were measured by qRT-PCR. **(F)** Predicted interaction between miR-218 and the two putative binding sites in the SLAM 3'UTRs. **(G)** Dual-luciferase assay of HEK293T cells transfected with luciferase constructs containing the two putative miR-218-binding sites (binding site 1 or binding site 2) together with synthetic mature miR-218 or a synthetic control miRNA with a scrambled sequence (miR-C). **(H)** Dual-luciferase assay of HEK293T cells transfected with luciferase constructs containing the wild-type 3′UTRs (3′WT UTRs) or a mutated 3′UTRs (with deletion of the miR-218-responsive element) from SLAM, plus miRNA. The levels of SLAM were normalized to the levels of β-actin. The levels of miR-218 were normalized to the levels of 5S. Results are expressed as means ± standard deviation (SD) from three independent experiments. *P*-values were calculated using Student's t test. An asterisk indicates a comparison with the indicated control. *P < 0.05, **P < 0.01.

To determine whether miRNA has emerged as an important component in the modulation of SLAM expression, we initially assessed miRNA expression profile in goat PBMCs infected with PPRV for 24 h vs. mock infected cells by using small RNA deep sequencing. This time postinfection has been demonstrated to show maximal changes in SLAM expression relative to uninfected cells both in the current study and previously studies (31, 32). Next, we identified differentially expressed miRNAs that were predicated by TargetScan to target the SLAM family genes and their fold change. Among these miRNAs, we found seven miRNAs target to SLAM family members, including SLAM, SLAMF5, SLAMF6, SLAMF7, SLAMF8, and SLAMF9 (Table 1 and Figure 2A). We focused on miR-218 because it was identified to target to SLAM and the expression of miRNA-218 in PPRV-infected cells decreased significantly relative to mock-infected cells (Table 1 and Figure 2A). To confirm this result, the kinetics of miR-218 and SLAM mRNA expression in PBMCs during PPRV infection were analyzed by qRT-PCR. Collectively, an inverse correlation between the expression of miR-218 and SLAM mRNA following PPRV infection was confirmed either in a post infection time-dependent (Figures 2B,C) or in a virus dose-dependent manner (Figures 2D,E). Two putative binding sites contained in the SLAM 3′ UTRs were further identified by using the TargetScan algorithm (Figure 2F). To confirm that one or both predicated binding sites were functional, the fragment of the SLAM 3′UTRs mRNA (NM_174184.4) containing putative miRNA binding sites was cloned into the psiCHEK-2 vector, then, these constructed vectors were transfected into HEK293 cells along with synthetic mature miR-218 (miR-218 mimic, miR-218) or a scrambled sequence control miRNA (MC). The results showed that miR-218 significantly inhibited the relative luciferase activity of the binding site 2 contained vector in comparison with control. However, no inhibitory effect was detected on the activity of binding site 1 (Figure 2G). Furthermore, the inhibitory effect was abrogated in cells transfected both with miR-218 and *SLAM* 3′UTRs containing mutated binding site 2 (Figure 2H).

**Table 1:** Differentially expressed cellular microRNAs predicted targets SLAM family members in PPRV- vs. Mock-infected goat PBMCs.

**miRNA name**	**Sequence (5^**′**^-3^**′**^)**	**Up down regulation**	**Fold change in expression**	**Target SLAM family members gene**
chi-miR-423-5p	ugaggggcagagagcgagacuuu	DOWN	−4.056	SLAMF8
chi-miR-877-5p	guagaggagauggcgcagggg	DOWN	−3.713	SLAMF7
novel_mir3	cacgcucaugcacacacccac	DOWN	−3.233	SLAMF6,SLAMF8, SLAMF9
novel_mir328	ggcagagaggaaagaggcucgg	UP	3.156	SLAMF5
chi-miR-374a-5p	uuauaauacaaccugauaagu	UP	−2.269	SLAMF8
chi-miR-1	uggaauguaaagaaguauguau	DOWN	−8.269	SLAMF5, SLAMF8
novel_mir218	cucccagcgcugucaccac	DOWN	−6.09	SLAM, SLAMF6

### miR-218 Regulates SLAM Expression and Virus Replication in PPRV Infected Goat PBMCs

**Figure 3:**
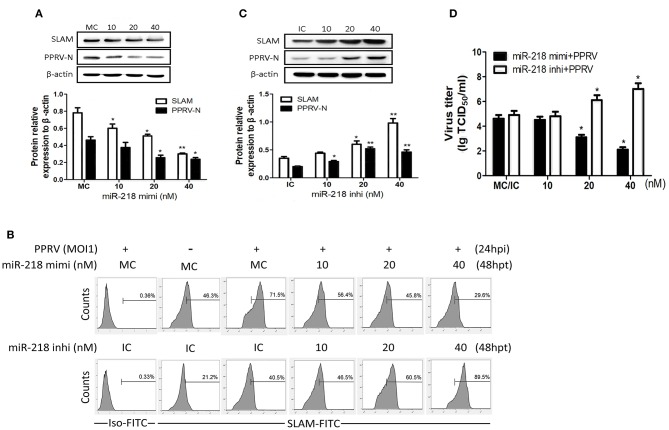
miR-218 regulates SLAM expression and virus replication in PPRV-infected goat PBMCs. **(A)** Goat PBMCs were transfected with different concentrations of miR-218 mimic or control mimic (MC), and 48 h later the cells were infected with PPRV at an MOI of 1. Twenty-four hours later, the protein levels of SLAM, and PPRV-N expression were measured by Western blot. **(B)** Goat PBMCs were transfected with miR-218 inhibitor, miR-218 mimic, or respective controls, and 48 h later the cells were infected with PPRV at an MOI of 1. Twenty-four hours later, cell-surface expression of SLAM on goat PBMCs was measured by flow cytometry. **(C)** Goat PBMCs were transfected with different concentrations of miR-218 inhibitor or IC, and 48 h later the cells were infected with PPRV at an MOI of 1. Twenty-four hours later, the protein levels of SLAM and PPRV-N expression were measured by Western blot. **(D)** Goat PBMCs were transfected with miR-218 inhibitor, miR-218 mimic, or respective controls, and 48 h later the cells were infected with PPRV at an MOI of 1. Twenty-four hours later, the virus titers in the supernatants were measured by TCID_50_ assay. β-actin was used as a loading control in Western blot analysis. Results are expressed as means ± standard deviation (SD) from three independent experiments. *P*-values were calculated using Student's t test. An asterisk indicates a comparison with the indicated control. *P < 0.05, **P < 0.01.

Screening for miRNAs with RNAhybrid and TargetScan tools identified miR-218 is a strong candidate repressor of the *SLAM* gene. Therefore, subsequent study was focused on miR-218. To test this miRNA, miR-218 mimic, or miR-218 inhibitor was transfected into goat PBMCs and 48 h later the cells were infected with PPRV at an MOI of 1. After 24 h infection of PPRV, the expression of SLAM was examined by Western blot and flow cytometry. We observed that pretreatment with increasing concentrations of miR-218 mimic caused reduced SLAM protein expression levels (Figure 3A) and the percentage of SLAM-positive cells (Figure 3B) in a dose-dependent manner as compared with that of control mimic (MC). To further ascertain that SLAM upregulation is the result of miR-218 downregulation at early PPRV infection, PBMCs were pretransfected with miR-218 inhibitor for 48 h followed by PPRV infection for 24 h. Results showed that, compared with control inhibitor (IC), transfection with increasing concentrations of miR-218 inhibitor led to enhancement of SLAM protein levels (Figure 3C) and the percentage of SLAM-positive cells (Figure 3B) in a dose-dependent manner.

It has previously been shown that the expression levels of SLAM have high correlation with replication of PPRV in goat PBMCs (18). In the present study, Western blot and TCID_50_ analysis showed that miR-218 mimic downregulated the levels of PPRV N protein (Figure 3A) and the virus titers (Figure 3D) in a dose-dependent manner, while miR-218 inhibitor increased the levels of PPRV N protein (Figure 3C) and the virus titers (Figure 3D) in a dose-dependent manner. Altogether, these results clearly showed that cellular miR-218 have negative effects on SLAM expression and PPRV replication.

### miR-218 Regulates SLAM Mediated Cytokines Production in PPRV-Infected Goat PBMCs

**Figure 4:**
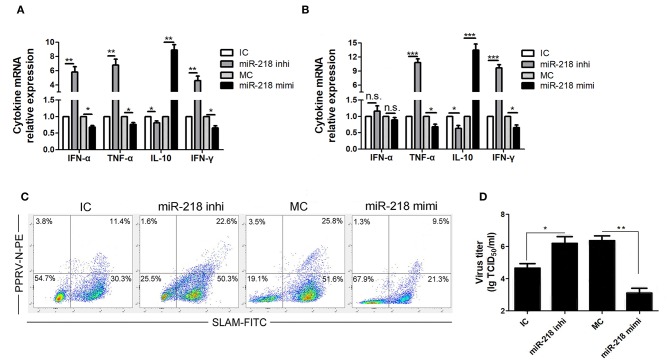
miR-218 regulates SLAM mediated immune responses in PPRV-infected goat PBMCs. **(A,B)** Goat PBMCs were transfected with control inhibitor (IC), miR-218 inhibitor, control mimic (MC), or miR-218 mimic for 48 h, and then the cells were mock infected **(A)** or infected with PPRV **(B)** at an MOI of 1. Twenty-four hours later, the mRNA levels of cytokines indicated were examined by qRT-PCR. **(C)** Goat PBMCs were transfected with IC, miR-218 inhibitor, MC, or miR-218 mimic, and 48 h later the cells were infected with PPRV at an MOI of 1. Twenty-four hours later, SLAM cell surface expression and PPRV-N positive cells were analyzed by flow cytometry. **(D)** Goat PBMCs were transfected with IC, miR-218 inhibitor, MC, or miR-218 mimic, and 48 h later the cells were infected with PPRV at an MOI of 1. Twenty-four hours later, the virus titers in the supernatants were measured by TCID_50_ assay. Results are expressed as means ± standard deviation (SD) from three independent experiments. *P*-values were calculated using Student's t test. An asterisk indicates a comparison with the indicated control. *P < 0.05, **P < 0.01.

Because PPRV-induced immunosuppression may attribute to the modulation of SLAM signaling in lymphocytes, the effect of SLAM expression on the production of various cytokines by PBMCs in the absence or presence of PPRV was determined. The cells were first transfected with miR-218 inhibitor, miR-218 mimic, or respective controls, followed by PPRV infection (MOI = 1). At 24 h post infection, the change in transcription of mRNA for various cytokines was determined by real-time PCR assays. We found that, in the absence of PPRV, miR-218 inhibitor stimulated IFN-α, TNF-α, IFN-γ expression, and suppressed IL-10 expression compared to inhibitor control, while a decreased IFN-α, TNF-α, IFN-γ, and increased IL-10 expression were detected in miR-218 mimic group compared to mimic control (Figure 4A). Interestingly, PPRV infection enhanced the change extent of detected cytokines either in miR-218 inhibitor or in miR-218 mimic group as compared to that in the absence of PPRV (Figure 4B). No obvious changes of IFN-α expression were detected in PBMCs transfected with miR-218 inhibitor, miR-218 mimic, or respective controls in the presence of PPRV (Figure 4B).

Since the expression levels of SLAM have high correlation with PPRV infection percentages in goat PBMCs (18), we also detected SLAM cell surface expression and PPRV positive cells in PPRV-infected cells pretransfected with miR-218 inhibitor, miR-218 mimic, or respective controls by FACS analysis. As show in Figure 4C, a significant increased percentage of SLAM positive cells were detected in miR-218 inhibitor transfection group compared to inhibitor control, while reduced percentage of SLAM positive cells was detected in miR-218 mimic transfection group compared to mimic control. Furthermore, the percentage of SLAM+PPRV+ cells in miR-218 inhibitor transfection group was significantly higher as compared with that in miR-218 mimic transfection group. Importantly, the percentage of SLAM+PPRV- cells in miR-218 inhibitor transfection group was significantly higher compared to miR-218 mimic transfection group (*P* < 0.05).

Moreover, in order to investigate the role of SLAM-mediated the immune responses in the regulation of PPRV production, the virus titers were evaluated by TCID_50_ assay. The results showed that miR-218 inhibitor transfection facilitated the production of PPRV as compared to inhibitor control (Figure 4D), while miR-218 mimic transfection caused a significant reduction in viral production in PBMCs as compared with mimic control (Figure 4D).

### Replication of PPRV Is Required for Inhibition of miR-218 Expression

**Figure 5:**
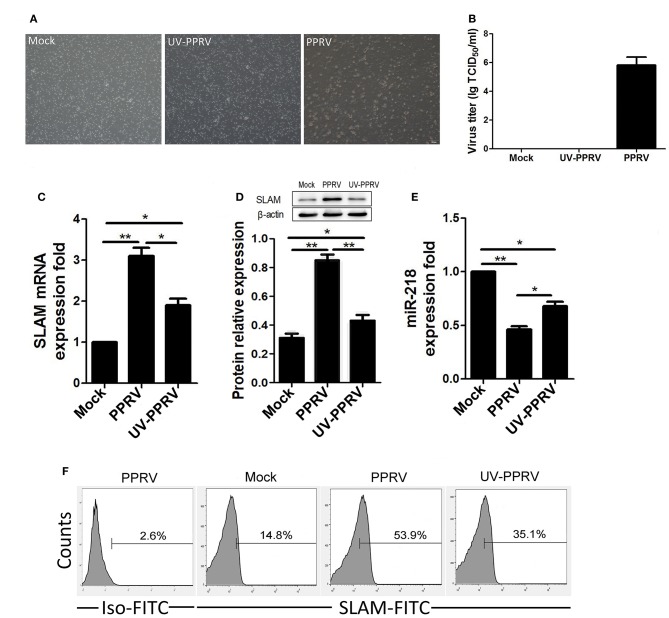
The replication of PPRV is required for inhibition of miR-218 expression in goat PBMCs. **(A,B)** Goat PBMCs were mock infected, infected with PPRV, or with UV-inactivated (UV-PPRV) at an MOI of 1 for 24 h, and cytopathic effect (CPE), and virus titers in PBMCs was measured by phase contrast microscope **(A)** and TCID_50_ assay **(B)**, respectively. **(C–E)** Goat PBMCs were mock infected, infected with PPRV, or with UV-inactivated (UV-PPRV) at an MOI of 1 for 24 h, and the intracellular expression levels of SLAM were measured by qRT-PCR **(C)**, and Western blot **(D)**. β-actin was used as a loading control in Western blot analysis. **(E)** Goat PBMCs were mock infected, infected with PPRV, or with UV-PPRV at an MOI of 1 for 24 h, and the levels of miR-218 were detected by qRT-PCR. The levels of miR-218 were normalized to the level of 5S. **(F)** Goat PBMCs were mock infected, infected with PPRV, or with UV-PPRV at an MOI of 1 for 24 h, and cell-surface expression of SLAM on goat PBMCs was measured by flow cytometry. Results are expressed as means ± standard deviation (SD) from three independent experiments. *P*-values were calculated using Student's t test. An asterisk indicates a comparison with the indicated control. *P < 0.05, **P < 0.01.

To explore whether viral replication play a role in miR-218-mediated SLAM expression in PPRV-infected PBMCs, the capability of ultraviolet (UV) irradiation inactivated live PPRV for modulation of miR-218 and SLAM expression was determined. As shown in Figures 5A,B, a clear cytopathic effect (CPE) and significant virus titers in active PPRV infected goat PBMCs were detected while not in UV-inactivated PPRV infected cells. Furthermore, the expression of SLAM mRNA was upregulated by 3.2-fold in response to PPRV infection compared to mock infection, while a 1.8-fold increase in SLAM expression was observed in UV-PPRV infected cells (Figure 5C). Similar results were obtained by Western blot analysis (Figure 5D). These results were also confirmed by flow cytometry analysis (Figure 5F). Taken together, these results indicated that SLAM expression by UV-PPRV infection was not completely inhibited compared to mock infection group. We further confirmed that the expression of miR-218 has inverse correlation with SLAM mRNA expression in mock-, PPRV-, and UV-PPRV-infected cells (Figure 5E).

### The Expression of PPRV H Protein Is Sufficient to Regulate miR-218-Mediated SLAM Expression

**Figure 6:**
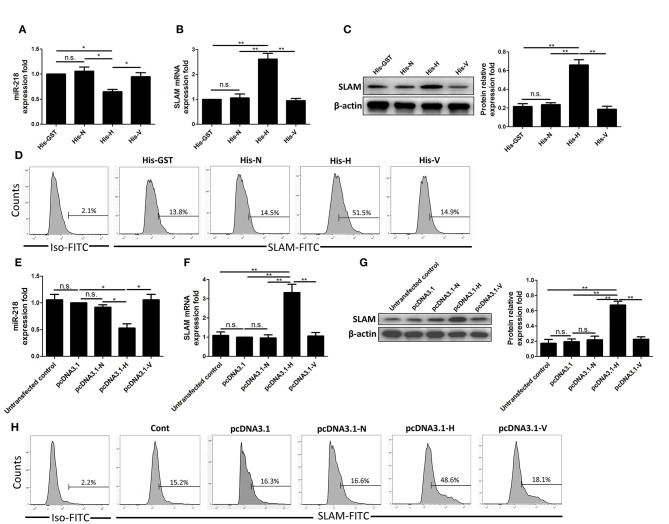
The expression of PPRV H protein is sufficient to regulate miR-218-mediated SLAM expression. His-tagged GST, PPRV-V, PPRV-H, and PPRV-N expressed in *E. coli* BL-21 and purified in Ni-NTA columns. The purified products were subjected to Western blot with anti-His antibody. His- GST used as a control. **(A)** Goat PBMCs were incubated with 100 ng/mL His-GST, His-N, His-H, and His-V for 24 h, and the miR-218 expression was subjected to qRT-PCR analysis. **(B–D)** Goat PBMCs were incubated with 100 ng/mL His-GST, His-H, and His-N for 24 h, and the expression of SLAM was subjected to qRT-PCR **(B)**, Western blot **(C)**, and flow cytometry analysis **(D)**. Goat PBMCs were transfected with pcDNA3.1-HA, pcDNA3.1-H-HA, pcDNA3.1-N-HA, and pCDNA3.1-V-HA plasmid for 48 h and cell lysates from transfected and untransfected control cells were subjected to Western blot with antibody against HA. **(E)** Goat PBMCs were transfected with pcDNA3.1-HA, pCDNA3.1-H-HA, pCDNA3.1-N-HA, and pCDNA3.1-V-HA plasmid for 48 h, and miR-218 expression in transfected and untransfected control cells was subjected to qRT-PCR analysis. **(F–H)** Goat PBMCs were transfected with pcDNA3.1-HA, pCDNA3.1-H-HA, pCDNA3.1-N-HA, and pCDNA3.1-V-HA plasmid for 48 h, and the expression of SLAM in transfected and untransfected control cells was subjected to qRT-PCR **(F)**, Western blot **(G)**, and flow cytometry analysis **(H)**. The levels of miR-218 were normalized to the level of 5S. β-actin was used as a loading control in the qRT-PCR and Western blot analysis for SLAM expression. Results are expressed as means ± standard deviation (SD) from three independent experiments. *P* values were calculated using Student's t test. An asterisk indicates a comparison with the indicated control. *P < 0.05, **P < 0.01.

PPRV H protein has previously been demonstrated to interact with SLAM receptor (19). To determine whether H by itself could cause miR-218-mediated up regulation of SLAM, the purified fusion protein His-GST, His-N, His-H, and His-V of PPRV were utilized in subsequent experiments. His-GST was used as controls. Goat PBMCs were incubated with His-GST, His-N, His-H, and His-V for 24 h, and the cells were harvested and SLAM and miR-218 expression was subjected to Western blot and qRT-PCR analysis, respectively. Analogous to our results with PPRV, H protein incubation resulted in the down regulation of miR-218 (Figure 6A). Furthermore, H protein caused the up regulation of mRNA (Figure 6B), protein (Figure 6C), and surface expression of SLAM (Figure 6D) on the cells, which indicates that PPRV H protein could regulated miR-218-mediated up regulation of SLAM in goat PBMCs. Additionally, we determined the role of endogenous PPRV H in regulating miR-218 and SLAM expression in goat PBMCs. To this end, PBMCs were transfected with pCDNA3.1-H-HA, pCDNA3.1-N-HA, and pCDNA3.1-V-HA plasmid, and transfected and untransfected control cells were subjected to Western blot with antibody against HA. The presence of the HA tag at the C terminus of H protein did not affect the protein's function (data not shown). Our data demonstrated that overexpression of H protein reduced miR-218 expression (Figure 6E) and up regulated SLAM expression in PBMCs (Figures 6F–H). Together, these experiments indicated that PPRV H protein alone was sufficient to cause the increase in the expression of SLAM receptor through down regulation miR-218 expression in goat PBMCs.

## Discussion

The tropism of the virus is closely associated with the expression of specific receptor on the cell surface (33, 34). PPRV is lymphotropic in nature and enters lymphoid cells through SLAM that is widely expressed on all immune cells (18, 19, 35). However, the regulation of SLAM expression in the ruminant and how this regulation affects viral replication is limited. Many viruses have been demonstrated to hijack the cellular miRNA to facilitate their replication (23, 24, 36). Here, we showed that PPRV modulate miRNA-218 expression in goat PBMCs and demonstrated the direct regulation of a cellular target, SLAM, by miR-218. We further demonstrated that expression of H protein of PPRV alone is sufficient for this effect. Thus, modulation of the miRNA-218 expression may play a key role in the pathogenesis of PPRV and viral replication.

Although it has been reported that the SLAM receptor on the cell surface appears to be downregulated by MV infection or by expression of MV hemagglutinin (H) protein (34, 37), the expression of SLAM in ruminant in responses to PPRV infection is not yet fully understood. Previous studies have demonstrated that PPRV vaccine strain Sungri/96 infection induced transient increased SLAM expression in goat PBMC at 48 hpi (31). Similar results were obtained in this study that upregulated SLAM expression in the goat PBMCs infected with PPRV vaccine Nigeria/75 (N75) strain at 24 hpi were observed. The contradictory findings about SLAM expression in response to PPRV and MV infection may due to the host-, cell-type or virus-derived factors. Studies have revealed that miRNAs could regulate viral infectivity either by targeting cellular factors essential for virus replication or by directly targeting virus genomes (23, 24). Recently, we showed that PPRV modulate goat PBMCs miRNA expression profile (26), which similar with the results obtained from studies that the miRNAs profiles changed in PPRV infected lung and spleen tissues of goats (25). To date, the role of miRNA plays in regulation of PPRV cellular receptors has not been considered. Here, we focused on miR-218 because it was predicated to target the *SLAM* gene, and more importantly, because the expression of miR-218 has an inverse correlation with SLAM expression during PPRV infection in a viral dose- or post infection time-dependent manner. It is important to note that sharply increased SLAM expression observed in PBMCs in response to PPRV at 24 hpi, may suggest that SLAM serve as an entry receptor for PPRV during early infection (31, 32). Since PPRV infection caused a rapid increase in SLAM cell surface expression at 24 hpi, we also detected SLAM expression in individual infected and non-infected cells by FACS and immunofluorescence analysis at 24 hpi. Surprisingly, although most PPRV-infected cells expressed SLAM receptor, a significantly increased SLAM expression on non-infected cells were also observed in PPRV infection group as compared with mock infection group. This imply that PPRV infected cells may contribute to the regulation of SLAM receptor expression on adjacent cells via intercellular communication. Exosomes have recently been characterized as bioactive vesicles that function to promote intercellular communication. Although exosomes and their contribution to replication and pathogenesis of viruses remain largely unexplored, a number of RNA viruses have been investigated in the field, including PRRSV, HBV, HCV, and Dengue virus (36, 38–40). The exosomes derived from virus infected cells containing altered composition confers numerous novel functionalities (38–40). Further study is necessary to understand the role of exosome pathway in regulation SLAM expression.

To obtain further evidence for the targeting of SLAM by miR-218, we examined whether putative binding sites predicted by TargetScan in the SLAM 3′UTRs were functional using luciferase reporter assay. Our results demonstrated that miR-218 can downregulate SLAM expression through directly targeting its mRNA. In addition, our data demonstrated miR-218 mimics suppressed the production of PPRV while miR-218 inhibitor had opposite effects. Previous studies have shown that the levels of SLAM expression have high correlation with PPRV replication in goat PBMCs (18). Here, our data clearly showed that miR-218 affects PPRV replication by regulating SLAM receptor expression.

SLAM signaling has been reported to function as a modifier in immunodeficiency disease (13–15). SLAM is a self-ligand receptor expressed on the activated lymphocytes, macrophages, and dendritic cells. Previous *in vitro* experiments suggested that SLAM/SLAM interactions stimulated inflammatory cytokines production and plays an important role in T-helper 1 (Th1) differentiation (41). Although SLAM signaling has been demonstrated to mediate measles virus induced immunosuppression (9), multiple SLAM-independent signaling pathways are involved in PPRV induced immunosuppression (42, 43). Our data clearly showed that SLAM expression modulate IFN-γ, TNF-α, and IL-10 mRNA expression in mock infected cells. Surprisingly, these modulatory effects were enhanced in the presence of PPRV infection. These results suggest that SLAM signaling may also contribute to PPRV induced immunosuppression.

To determine whether the replication of PPRV is required for the inhibition of miR-218 levels, we infected PBMCs with either activated or inactivated PPRV and then examined their effects on the miR-218 and SLAM expression. Although an inverse correlation between miR-218 expression and SLAM mRNA levels was observed in both groups, only part of SLAM expression lost in UV-inactivated PPRV group as compared with replication-component PPRV infected group. These results suggest that active PPRV replication is in part mediated the expression of SLAM, the binding of virus with its receptor may also contribute to miR-218-mediated the modulation of SLAM expression in goat PBMCs.

It has previously been shown that PPRV H protein can interact with SLAM receptor based on homology model of the complex (19). Here, our data showed that incubation of PBMCs with PPRV H protein alone can up regulate the protein expression and cell surface expression of SLAM on cells. Furthermore, in pCDNA3.1-H-HA transfected cells, similar results were obtained that expressed PPRV H protein regulating miR-218 and SLAM expression in goat PBMCs. This suggests that PPRV H protein plays an important role in miR-218 mediated SLAM up regulation during PPRV infection. In combined with the results that UV irradiation inactivated PPRV infection only induce part of SLAM expression as compare with replication-component PPRV infection group, these results may suggest that both the binding of PPRV H protein with SLAM on the surface of PBMCs and live virus replication contribute to miR-218 mediated the modulation of SLAM expression during PPRV infection.

In conclusion, our findings here suggest that cellular miR-218 contribute to regulate PPRV infectivity, through post-transcriptional regulation of SLAM receptor. Further study is necessary to understand the precise mechanism how miR-218 is regulated during PPRV infection and promoting virus replication.

## Data Availability

All datasets generated for this study are included in the manuscript/supplementary files.

## Ethics Statement

All animal experimental protocols were approved by the Northwest A&F University Animal Care Committee and followed the Guide for the Care and Use of Laboratory Animals published by the Chinese National Institutes of Health. The research protocols were conducted in accordance with the animal behavioral guidelines, using approved protocols from the institutional animal care committee (2014BAD23B11).

## Author Contributions

XQ and TW performed the majority of experiments. ZL, YZ, and BY participated part of the experiments. XQ and JW conceived the study, participated in its design, and coordination. XQ prepared the manuscript. All authors have read and approved the final manuscript.

### Conflict of Interest Statement

The authors declare that the research was conducted in the absence of any commercial or financial relationships that could be construed as a potential conflict of interest.
